# Effect of repeated-sprints on the reliability of short-term parasympathetic reactivation

**DOI:** 10.1371/journal.pone.0192231

**Published:** 2018-02-06

**Authors:** Matteo Bonato, Andrea Meloni, Giampiero Merati, Antonio La Torre, Luca Agnello, Gianluca Vernillo

**Affiliations:** 1 Department of Biomedical Sciences for Health, Università degli Studi di Milano, Milan, Italy; 2 IRCCS Fondazione Don Carlo Gnocchi, Milan, Italy; 3 IRCCS Istituto Ortopedico Galeazzi, Milan, Italy; 4 Human Performance Laboratory, Faculty of Kinesiology, University of Calgary, Calgary, Canada; University of Bourgogne France Comté, FRANCE

## Abstract

This study determined the reliability of post-exercise heart rate recovery (HRR) and vagal-related HR variability (HRV) after repeated-sprints (RSs), and contrasted it with the smallest worthwhile change (SWC) of these indices. Fourteen healthy male participants performed on four occasions, separated by 7 days, five 30-m sprints interspersed by 25-s of recovery. Post-exercise HR during 10 min of seated rest was measured. HRR during the first 60-s of recovery was computed (HRR_60s_). HRV indices were calculated in time and frequency domains during the last 5-min of the recovery. Absolute and relative reliability were assessed by typical error of measurement expressed as coefficient of variation (CV) and intraclass correlation coefficients (ICCs), respectively. Sensitivity was assessed comparing SWC to the typical error of measurement. CV ranged from 3.6% to 13.5% and from 6.3% to 109.2% for the HRR and HRV indices, respectively. ICCs were from 0.78 to 0.96 and from 0.76 to 0.92, respectively. HRR and HRV indices showed large discrepancies reliability. HRR_60s_ and the square root of the mean sum of the squared differences between R-R intervals presented the highest levels of both absolute and relative reliability. However, SWC was lower than the typical error of measurement, indicating insufficient sensitivity to confidently detect small, but meaningful, changes in HRR and HRV indices.

## Introduction

Repeated-sprints exercise (RSE) is characterized by short-duration sprints at supramaximal intensities interspersed with brief recoveries [1,2]. Through the years, RSE has piqued the interest of the scientific community because it may offer a viable alternative to classically prescribed aerobic training protocols and it is therefore considered a reliable way to induce metabolic adaptations in human skeletal muscle [1,2]. Despite the time course of postexercise heart rate (HR) recovery (HRR) and variability (HRV) is acutely impaired after RSE [3–5], our group recently observed that 8-weeks of RS training might have a significant and positive effect on the short-term post exercise parasympathetic reactivation [6]. Indeed, in nine healthy participants who performed 18 maximal all-out 15-m sprints interspersed with 17 s of passive recovery, 3 times a week for 8 weeks, postexercise HRR and HRV indices improved when compared to a control group that performed normal, daily physical activities [6].

The evaluation of the time course of postexercise HRR and HRV is considered an accurate non-invasive method to monitor training status. This assessment reflects the general hemodynamic adjustments in relation to body position, blood pressure regulation and meta-baroflex activity, which partly drives sympathetic withdrawal and parasympathetic reactivation and it has extensively been used to predict exercise performance and cardiovascular recovery after training [7,8]. Indeed, the autonomic nervous system may play a role in the training response [9] and it may further provide information regarding the physiological adaptation occurring as a consequence of a given training stimulus [8]. This has been conducted at rest [10], during exercise [11] and throughout the recovery period [4,10,12]. Interestingly, resting cardiac parasympathetic activity is related to cardiorespiratory fitness [9,13,14]. Further, Buchheit [7] argued that measures of HRR and HRV can be used (i) to assess acute fatigue/recovery responses to isolated aerobic-oriented training sessions, and (ii) to inform on both positive and negative adaptations to aerobic oriented training blocks. Accordingly, the reliability (i.e. the degree of change in a particular measure when repeated on different occasions, but in similar conditions [15]) of HRR and HRV measurements as part of sport, physiological and clinical research is very important, in order to allow rigorous evaluation of adaptation to training programs [16] and, therefore, it has been frequently investigated. However, an adequate consensus has not been fully reached since studies on healthy populations have reported low-to-moderate reliability of HRR and HRV indices at rest and after submaximal or maximal exercises [17–21]. As such, results of reliability studies are heterogeneous, and dependent on a number of factors such as (i) the experimental intervention, (ii) the signal selection and analysis, and (iii) the statistical analysis used [16,21].

However, the reliability of HRR and HRV indices after an RSE has not been tested yet. We therefore reasoned that without prior knowledge of the between test reliability, interpretation of any information about changes in the cardiac parasympathetic reactivation after an RSE is limited. Indeed, despite we previously showed that changes in HRR and HRV indices could be detected after an RS training [6], and thus the smallest worthwhile change (SWC) could be interpreted as real within acceptable limits of probability [22,23], to assess the usefulness of parasympathetic reactivation measurements after RSE it is important to compare the SWC to the reliability data for the considered indices. This would be of great importance for strength and conditioning coaches, as well as practitioners, in order to avoid bias interpretation when assessing changes in the parasympathetic reactivation indices after RS training protocol and to be able to present a change as meaningful.

Since limited reliability and sensitivity data on parasympathetic reactivation after RSE are available, the main aim of this study was to establish the short-term reliability, as well as the sensitivity, of post-exercise parasympathetic reactivation measures after RSE.

## Methods

### Participants

Fourteen healthy moderately-trained males (mean ± SD; age: 24 ± 4 yrs, stature: 181 ± 5 cm, body mass: 74 ± 7 kg, BMI: 23 ± 1 kg·m^-2^, training 8 ± 2 h per week) volunteered to participate in this study. All participants were involved in various intermittent activities (e.g. basketball and soccer) and they were all familiar with exercise testing. None of the participants had any history or clinical signs of cardiovascular or pulmonary diseases. Each participant gave written informed consent prior to the study. The study was approved by the local ethical committee (protocol #58/15, Università degli Studi di Milano, Italy) and it was performed according to the ethical standards laid out inthe Helsinki Declaration for experimentation on human participants.

### Procedures

Participants completed on four occasions, separated by 7 days, an RSE. They were asked to refrain from intense physical activity in the 48-h preceding each testing session. In order to minimize circadian effects, all RSEs were conducted at same hour of the day (± 15 min). All participants were allowed to maintain their usual diet throughout the study, but were asked to consume their last meal at least 3 h before each test session and to refrain from consuming drinks containing caffeine for at least 12 h preceding the effort. Smokers were not included in the present study. Further, water consumption was restricted at the start of each RSE since it has been shown that water intake accelerates post-exercise cardiac vagal reactivation [24].

### Repeated-sprints exercise

RSE was performed on an indoor synthetic track where the ambient temperature ranged from 18 to 20°C. The RSE consisted of five 30-m linear sprints interspersed by 25 s of active recovery [6]. To avoid any protective pacing strategy, each subject completed a preliminary single sprint that was considered as the criterion score during the RSE. Specifically, if the performance time in the first sprint of the RSE was worse than the criterion score (i.e. > 2.5%), the test was immediately terminated and participants were required to repeat the RSE with a higher effort after a 5-min rest [25]. Five seconds before starting each sprint, the participants were told to assume the ready position, that was standardized for each subject throughout the protocol [26], and to wait for the start signal, which was announced by a countdown given by the experimenter. Strong verbal encouragement was provided to each participant during all sprints. An electronic twin-beam photocell system (Microgate, Bolzano, Italy) was mounted at a fixed height (1.2 m from the floor [27]) to record each RS with a precision of 0.01 s. The sum of times for all sprint repetitions [2] and the percentage sprint decrement [S_dec_, 100—(total time/ideal time × 100), ideal time = 5 × best time] were then calculated. S_dec_ is considered the most valid and reliable method to quantify fatigue in RS testing [28].

### Beat-to-beat HR analysis

#### Materials and data treatment

A Polar S810 HR monitor (Polar Electro, Kempele, Finland) continuously recorded beat-to-beat HR at a 1-ms time resolution [29] by means of an electrode transmitter belt (T61, Polar Electro) that was fitted to the chest of each subject after application of conductive gel. All R-R series recorded by the HR monitor were extracted on a personal computer with the processing program (Polar precision performance SW 5, Polar Electro). Occasional ectopic beats were visually identified and manually replaced with interpolated adjacent R-R interval values.

### Postexercise HRR assessment

Parasympathetic nervous system reactivation was assessed during the 10-min period after the RSE. On completing the RSE, the participants were immediately asked to sit on a chair. Time interval between the end of the RSE and sitting down was < 5 s. Particular attention was paid to this detail because differences in body posture have been shown to result in different absolute HRR values [30]. Peak HR (HR_peak_) was considered as the highest RR-interval value reached at the end of the RSE. As previously described [6], HRR was calculated by computing the absolute difference between the peak HR retained at the end of exercise (mean of 5 s—HR_peak_) and the HR recorded following 60 s of recovery (HRR_60s_) and by taking the time constant of the HR decay obtained by fitting the 10-min postexercise HRR into a first-order exponential decay curve (HRR_τ_).

### Short-term resting HRV analysis

HRV analyses of the last 5 min of the 10-min recovery period with the subject in the sitting-resting position were performed to ensure data stability. We did not control the respiratory rate in this study sample to not perturb the natural return of HR to baseline and to enhance the applicability of our results in the field [31]. However, the respiratory rate was always within the HF range (> 0.15–0.40 Hz) and did not differ significantly during the last 5 min of recovery [3]. Further, vagal-related HRV indices during spontaneous and metronome-guided breathing differ little [32] and do not seem to influence the reliability of HRV indices [33], though no unanimous consensus exists [34]. As previously described [6], HRV values were restricted to the natural logarithm of the (i) square root of the mean sum of the squared differences between R-R intervals (Ln rMSSD_5-10min_); (ii) standard deviation of R-R intervals (Ln SDNN_5-10min_); (iii) high frequency power (Ln HF_5-10min_), and (iv) percentage of change in successive normal sinus (NN) intervals with increases lower than 50 ms (pNNx^+^_5-10min_) since pNNx with x < 50 ms is more sensitive to characterise cardiac response to exercise and provides useful information about the short-term control of sinus rhythm dynamics [35]. All analyses were performed with Kubios HRV Analysis Software v2.2 (Biosignal analysis and medical imaging group, University of Eastern Finland, Finland) [36], excepted for pNNx^+^_5-10min_ for which a customized software was used (LabVIEW National Instruments, Version 7.1, Austin, TX).

### Statistical analysis

Data are presented as mean ± standard deviation, 90% confidence interval, and range. To examine the reliability of RSE over the 4 consecutive trials, systematic changes in the mean across the RSE sessions (i.e. RS 1, RS 2, RS 3, and RS 4) were primarily assessed using a repeated-measures ANOVA, with the Bonferroni post-hoc test procedures and the level of significance sat at *P* < 0.05. Sphericity was checked by Mauchly’s test and *P* values were modified with Greenhouse-Geisser or Huyn-Feldt corrections if necessary. The analysis was carried out with IBM^TM^ SPSS^TM^ Statistics (version 22.0.0, IBM Corp., Somers, New York, USA). Then, relative and absolute reliability was assessed using the Intraclass Correlation Coefficient (ICC) and the typical error of measurement as the coefficient of variance (CV), respectively [37]. Relative reliability represents the degree to which individuals maintain their position in a sample with repeated measurements. Absolute reliability is the degree to which repeated measurements vary for individuals [38]. We used a specifically designed spreadsheet (http://sportssci.org) that provides reliability statistics for consecutive pairs of trials for each participant when there are at least 2 trials. In this spreadsheet, all analyses were performed on log-transformed data to reduce bias arising from non-uniformity of error [11]. The sensitivity (i.e. signal to noise ratio) was established comparing the smallest worthwhile change (0.2 × between participant’ standard deviation) with typical error of measurement [22] associated with RS 1, RS 2, RS 3, and RS 4.

## Results

### RSE

**Fig 1:**
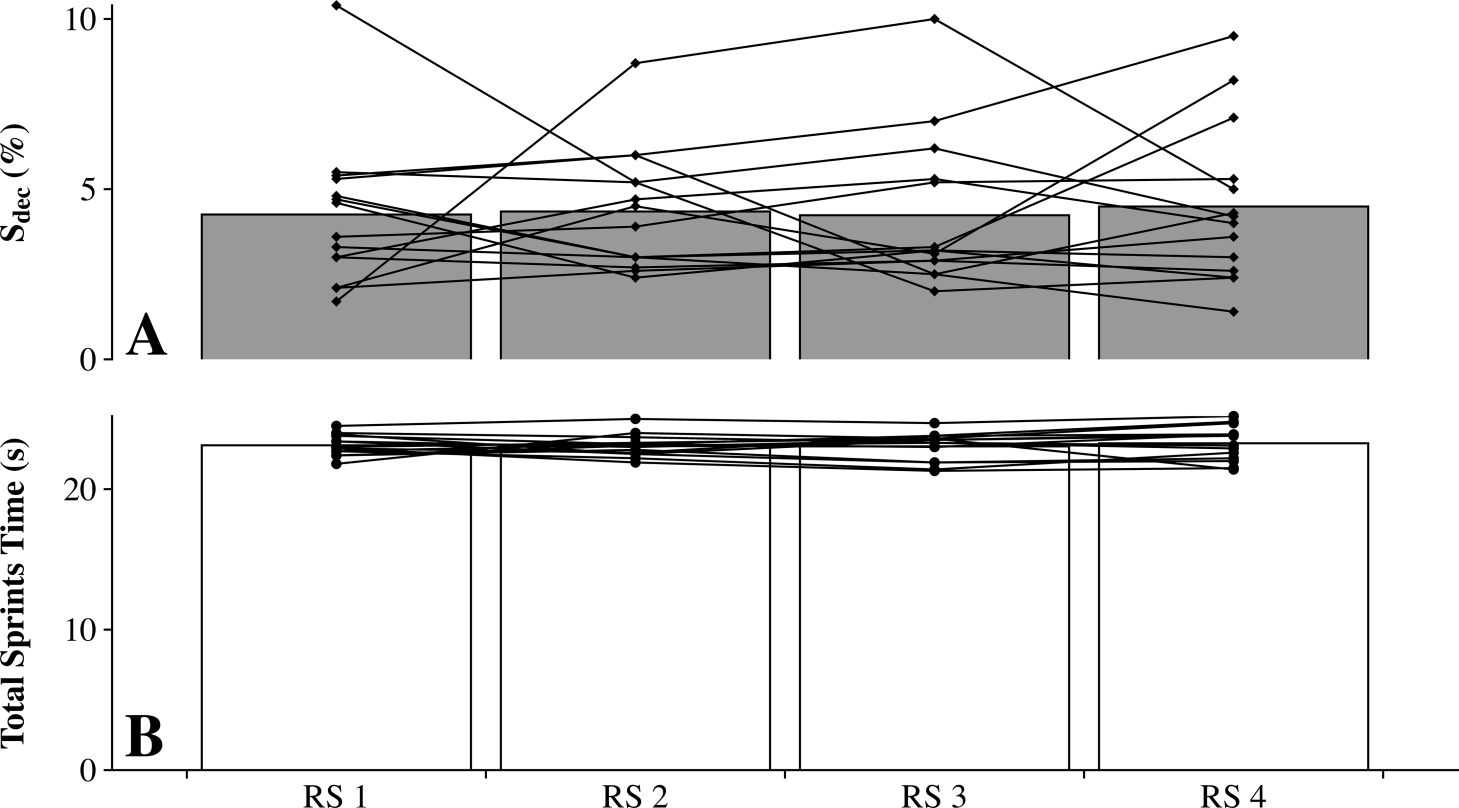
Performance in the repeated-sprint exercise. Performance is expressed as percentage sprint decrement (S_dec_, Panel A) and total sprints time (Panel B) for the 14 participants on four RS sessions separated by one week (i.e. RS 1, RS 2, RS 3, and RS 4). Mean and individual values are also presented.

S_dec_ scores were 4.3 ± 2.2% (range: 1.7–10.4%), 4.3 ± 1.8% (2.4–8.7%), 4.2 ± 2.2% (2.0–10.0%) and 4.5 ± 2.4% (1.4–9.5%) (Fig 1A); whereas total sprints time were 23.1 ± 0.7 s (21.8–24.0 s), 23.1 ± 0.8 s (21.9–24.0 s), 23.0 ± 1.0 s (21.3–23.8 s) and 23.3 ± 21.2 s (21.4–24.8 s) for the RS 1, RS 2, RS 3 and RS 4 conditions, respectively (Fig 1B). There was no significant session effect for any of the measures (*P* > 0.05).

### Reliability of postexercise HRR and short-term resting HRV indices

HRR and HRV values over the 4 consecutive RSEs, as well as the sensitivity data, are reported in Table 1. There was no significant session effect for any of the measures (*P* > 0.05). The typical error exceeded the SWC for both the HRR and HRV indices.

**Table 1:** Mean ± SD of the heart rate recovery and variability indices on four RS sessions separated by one week (i.e. RS 1, RS 2, RS 3, and RS 4).

Index	RS 1	RS 2	RS 3	RS 4	Session effect (*P* level)	SWC	TE
***Heart Rate Recovery***							
HR_peak_ (beats·min^-1^)	173.5 ± 16.1	178.7 ± 10.2	179.1 ± 10.1	177.3 ± 10.3	0.46	2.37	5.88
HRR_60s_ (beats·min^-1^)	41.2 ± 12.4	43.6 ± 14.2	44.9 ± 12.3	46.3 ± 11.5	0.07	2.49	4.07
HRR_τ_ (s)	65.1 ± 21.0	71.3 ± 33.7	68.4 ± 24.2	68.6 ± 27.8	0.34	5.28	9.91
***Heart Rate Variability***							
Ln rMSSD_5-10min_ (ms)	2.16 ± 0.81	2.03 ± 0.64	1.94 ± 0.57	1.96 ± 0.57	0.61	0.13	0.27
Ln SDNN_5-10min_ (ms)	3.13 ± 0.45	3.16 ± 0.38	3.06 ± 0.40	3.06 ± 0.40	0.59	0.08	0.18
Ln HF_5-10min_ (ms)	2.91 ± 1.35	2.75 ± 1.12	2.60 ± 1.22	2.60 ± 1.21	0.67	0.24	0.46
pNN20^+^_5-10min_ (%)	3.9 ± 5.3	3.9 ± 5.2	3.2 ± 4.8	2.9 ± 3.8	0.48	0.94	1.72
pNN10^+^_5-10min_ (%)	10.2 ± 9.9	11.1 ± 9.8	10.1 ± 9.3	10.9 ± 8.1	0.81	1.81	3.8

Peak heart rate at the end of the exercise (HR_peak_); number of heart beats recovered in 60 s after exercise cessation (HRR_60s_); time constant of short-time heart rate recovery (HRR_τ_); natural logarithm of the square root of the mean sum of the squared differences between R-R intervals (Ln rMSSD_5-10min_); natural logarithm of the standard deviation of R-R intervals (Ln SDNN_5-10min_); natural logarithm of the high frequency power (Ln HF_5-10min_); percentage of change in successive normal sinus intervals with increases larger than 20 and 10 ms (pNNx^+^_5-10min_); smallest worthwhile change (SWC); typical error of measurement (TE). Subscript ‘5–10 min’ means calculated on the last 5 min of the recovery periods.

Reliability of HRR and HRV indices following RSEs is presented in Table 2. Mean ICC values range from 0.78 to 0.96 and from 0.76 to 0.92 for the HRR and HRV indices, respectively. Mean CV values from 3.6% to 13.5% and from 6.3% to 109.2%, respectively.

**Table 2:** Measures of relative [Intraclass Correlation Coefficient (ICC), (90% confidence interval)] and absolute [typical error of measurement as the coefficient of variance (CV), (90% confidence interval)] reliability of the heart rate recovery and variability indices on four RS sessions separated by one week (i.e. RS 1, RS 2, RS 3, and RS 4).

Index	ICC	CV (%)
***Heart Rate Recovery***		
HR_peak_	0.78 (0.61–0.90)	3.6 (3.0–4.6)
HRR_60s_	0.91 (0.82–0.96)	10.9 (9.0–14.2)
HRR_τ_	0.96 (0.91–0.98)	13.5 (11.1–17.6)
***Heart Rate Variability***		
Ln rMSSD_5-10min_	0.89 (0.80–0.95)	14.1 (11.6–18.5)
Ln SDNN_5-10min_	0.82 (0.67–0.92)	6.3 (5.2–8.2)
Ln HF_5-10min_	0.92 (0.85–0.97)	24.0 (19.7–31.8)
pNN20^+^_5-10min_	0.85 (0.69–0.93)	93.6 (69.2–148.0)
pNN10^+^_5-10min_	0.76 (0.57–0.89)	109.2 (85.5–160.6)

Peak heart rate at the end of the exercise (HR_peak_); number of heart beats recovered in 60 s after exercise cessation (HRR_60s_); time constant of short-time heart rate recovery (HRR_τ_); natural logarithm of the square root of the mean sum of the squared differences between R-R intervals (Ln rMSSD_5-10min_); natural logarithm of the standard deviation of R-R intervals (Ln SDNN_5-10min_); natural logarithm of the high frequency power (Ln HF_5-10min_); percentage of change in successive normal sinus intervals with increases larger than 20 and 10 ms (pNNx^+^_5-10min_). Subscript ‘5–10 min’ means calculated on the last 5 min of the recovery periods.

## Discussion

This study compared the relative and absolute reliability as well as the sensitivity of parasympathetic-mediated HR-derived indices after RSE. The results provide evidence that short-term reliability of post-exercise parasympathetic reactivation indices (i.e. HRR and HRV) showed large discrepancies in markers of reliability, in line with previous studies on HRR and HRV indices both at rest and after (sub)maximal exercises [17–21]. Further, based on the analysis of the sensitivity, TE consistently exceeded the SWC for both the HRR and HRV indices. Therefore, RSE does not provide sufficient sensitivity to assess training-induced changes in HRR and HRV indices after RSE.

The CV values of HRR_60s_ and HRV indices found in the present study showed good relative and absolute reliability. However, they were lower compared to what reported by Al Haddad et al. following supramaximal exercise [18]. This difference might be related to the different nature of the exercise protocols used. Indeed, Al Haddad et al. [18] used a Wingate test as supramaximal exercise that probably elicited a greater anaerobic contribution compared to our RSE model, resulting in a greater autonomic perturbation and, thus, in a worse reliability of the HRV indices.

Further, we assessed ICC as estimation of the percentage of the observed score variance that is attributable to the true score variance [37]. The higher the ICC, the higher the relative reliability, and the lower the influence of measurement error. The ICCs of HRR and HRV indices in the present study was in the range of the ICCs reported in other studies [17–21]. An interesting result was the high relative reliability of HRR_60s_ and Ln rMSSD_5-10min_ (Table 2), the main HRR index to monitor positive changes in high intensity exercises performance [39] and the more appropriate HRV index to monitor athletes in the field [7], respectively. However, the ICC does not provide an index of the expected trial-to-trial noise in the data; rather it reflects the ability of a measure to be differentiated between individuals [37]. Therefore, it was argued that the CV (i.e. the typical error of measurement as the coefficient of variance) seems to be the most appropriate measure of reliability, because it represents the noise occurring from trial-to-trial [15]. A more salient fact was the poor absolute reliability of both pNN20^+^_5-10min_ and pNN10^+^_5-10min_, though they are more sensitive indices to characterize cardiac response to exercise, providing useful information about the short-term control of sinus rhythm dynamics [35]. The fact that the pNNx family reflects the vagal modulation [35], that is highly impaired after RSE [3–5,40], probably contributes to the observed values.

However, CV as measure of absolute reliability does not give any information on whether the reliability is acceptable or not [23]. Indeed, the usefulness of a measurement is its ability to detect systematic changes at individual and group level [15]. This measurement characteristic can be evaluated comparing random changes (i.e. typical error of measurement representing the noise) with the smallest worthwhile change expected from a training intervention [22]. The difference between signal and noise defines the sensitivity in detecting systematic variation in training interventions [22]. In this study, the smallest worthwhile change of post-exercise HR measures showed to be not close enough to the typical error of measurement (Table 1). This indicates an unacceptable ability of the post-exercise HR measures to detect small but worthwhile variations at the individual level, likely due to the extremely high intensity of the RSE that is related to the persistent elevation of adrenergic factors and local metabolites during recovery (e.g. H+, lactate, Pi) [41], which impairs the parasympathetic activity [3].

## Conclusions

Though RSE seems to be an effective method to improve post-exercise parasympathetic function [6], it seems to be not associated with acceptable levels of signal stability during the recovery period, as already observed for submaximal exercises [18]. This is due to the noise in the measurement, suggesting caution when attempting to assess meaningful changes or differences in the post-exercise HR indices after longitudinal RSE interventions. Indeed, only when alterations in HRR and HRV exceed the typical error can practitioners confidently interpret a meaningful change in parasympathetic function after RSE.
